# ArrayExpress update – from bulk to single-cell expression data

**DOI:** 10.1093/nar/gky964

**Published:** 2018-10-24

**Authors:** Awais Athar, Anja Füllgrabe, Nancy George, Haider Iqbal, Laura Huerta, Ahmed Ali, Catherine Snow, Nuno A Fonseca, Robert Petryszak, Irene Papatheodorou, Ugis Sarkans, Alvis Brazma

**Affiliations:** 1 European Molecular Biology Laboratory, European Bioinformatics Institute, EMBL-EBI, Wellcome Trust Genome Campus, Hinxton, Cambridge CB10 1SD, UK; 2 CIBIO/InBIO-Centro de Investigação em Biodiversidade e Recursos Genéticos, Universidade do Porto, Rua Padre Armando Quintas, 4485-601 Vairão, Portugal

## Abstract

ArrayExpress (https://www.ebi.ac.uk/arrayexpress) is an archive of functional genomics data from a variety of technologies assaying functional modalities of a genome, such as gene expression or promoter occupancy. The number of experiments based on sequencing technologies, in particular RNA-seq experiments, has been increasing over the last few years and submissions of sequencing data have overtaken microarray experiments in the last 12 months. Additionally, there is a significant increase in experiments investigating single cells, rather than bulk samples, known as single-cell RNA-seq. To accommodate these trends, we have substantially changed our submission tool Annotare which, along with raw and processed data, collects all metadata necessary to interpret these experiments. Selected datasets are re-processed and loaded into our sister resource, the value-added Expression Atlas (and its component Single Cell Expression Atlas), which not only enables users to interpret the data easily but also serves as a test for data quality. With an increasing number of studies that combine different assay modalities (multi-omics experiments), a new more general archival resource the BioStudies Database has been developed, which will eventually supersede ArrayExpress. Data submissions will continue unchanged; all existing ArrayExpress data will be incorporated into BioStudies and the existing accession numbers and application programming interfaces will be maintained.

## INTRODUCTION

ArrayExpress is an archive of functional genomics data that includes a range of experiment types, such as gene expression, methylation profiling and chromatin immunoprecipitation assays. ArrayExpress was first established as a database for microarray data in 2002 (1) and for the last decade has been one of the core archival resources at the European Molecular Biology Laboratory, European Bioinformatics Institute (EMBL-EBI). ArrayExpress accepts submissions via the webtool Annotare and is the main source of data for the Expression Atlas (2) – a value-added gene expression database at EMBL-EBI, which allows for gene-, tissue- or disease-based queries. As one of the largest international databases providing stable access to experimental data and metadata, ArrayExpress has been recognized as a Core Data Resource by the European bioinformatics infrastructure project ELIXIR (https://www.elixir-europe.org/platforms/data/core-data-resources) since 2017.

As experimental technologies have changed, since 2008 ArrayExpress accepts functional genomics data from high-throughput sequencing technologies, in particular RNA sequencing (RNA-seq) experiments. Many of the new ArrayExpress developments since then have focused on optimizing our data submission and access interfaces to accommodate these types of data (3,4). During the last 12 months, for the first time the number of submissions from sequencing-based experiments have exceeded those from microarrays. For these experiments, the raw sequences are stored in the European Nucleotide Archive (ENA), whilst ArrayExpress retains any processed data, such as gene expression matrices, experimental metadata, e.g. what experimental variables have been tested in the experiment, as well as other metadata necessary for data re-use. For selected transcriptomics experiments, the data are consistently re-processed and re-annotated by our curation team and made available in Expression Atlas. A major shift over the last 2 years has been a rapid increase in data from experiments sampling single cells rather than mixtures of cells, namely those investigating RNA expression from single cells. As of August 2018, there are data from almost hundred single-cell based experiments in ArrayExpress.

Molecular biology experiments supporting experimental findings are becoming increasingly multi-faceted and typically employ a range of technologies, e.g. microarray-based genotyping with RNA-seq or proteomics assays. ArrayExpress was not designed to represent the interrelation of these technologically distinct datasets. Therefore a new generalized database, the BioStudies Database (5) has been developed at EMBL-EBI, to support the depositions of all data associated with a peer-reviewed publication. Over the next 2 years BioStudies will supersede ArrayExpress and all existing data will be made available through BioStudies' ‘ArrayExpress project’. The BioStudies backend will provide comparable data search and exploration capabilities as well as programmatic interfaces, making the transition as smooth as possible from the ArrayExpress user or submitter perspective.

## GROWTH OF SEQUENCING BASED DATA

**Figure 1.**
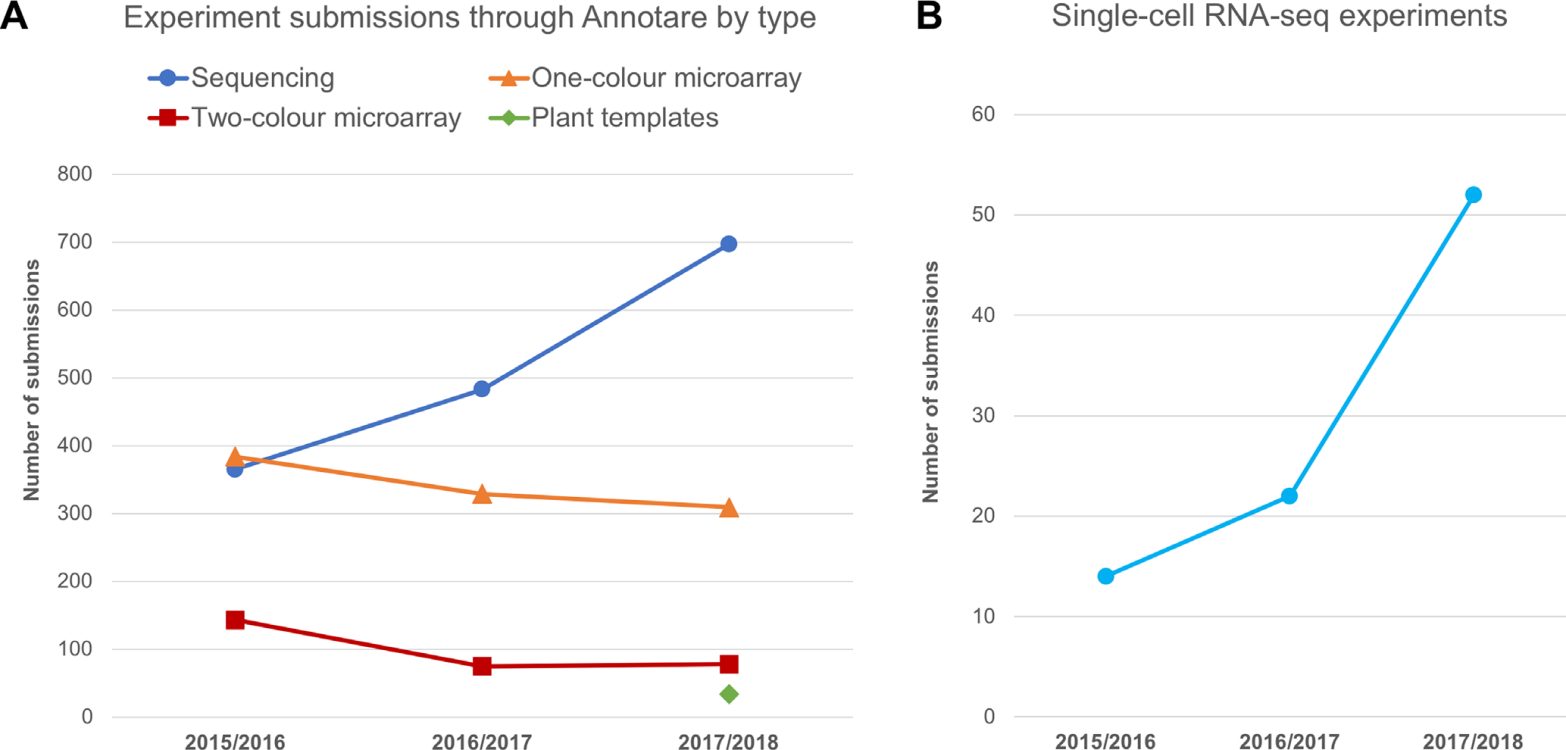
(**A**) Experiment submissions to ArrayExpress via Annotare during the last 3 years by experiment type. Data from September 2017 to August 2018 include 34 submissions using the newly introduced plant templates (27 plant sequencing, 5 plant one-colour microarray and 2 plant two-colour microarray experiments). (**B**) Number of experiments in ArrayExpress (public or private) with experiment type ‘RNA-seq of coding RNA from single cells’ by submission year.

ArrayExpress receives ∼1000 experiment submissions per year via the submission tool Annotare. Over the last year ∼700 of these were based on nucleotide sequencing (Figure 1A), with the highest proportion being RNA-seq experiments. Over the last 2 years there has also been a considerable increase in the number of submitted single-cell sequencing experiments (Figure 1B).

### Single-cell expression data and metadata

The first single-cell RNA-seq (scRNA-seq) experiment was submitted to ArrayExpress in 2011 (E-MTAB-609, the investigation of the transcriptome of MCF-7 breast cancer cells (6)). As of August 2018, ArrayExpress hosts 97 directly submitted scRNA-seq experiments; 72 have been released publicly (the rest are temporarily private, pending publication). The research questions investigated by scRNA-seq range from uncovering new cell types in whole mouse embryos, tracing the differentiation of embryonic or induced pluripotent stem cells, assessing the transcriptional response after viral infection to dissecting the heterogeneity of lung cancer specimens. Most experiments are performed in Mus musculus as a model organism or use human cell lines. The most frequently used scRNA-seq protocols currently are Smart-seq2 (7) and the droplet-based method by 10x Genomics.

Capturing sufficient metadata to ensure that each single-cell dataset is reproducible and the data can be re-analysed is a significant challenge. This becomes apparent when re-processing these data, for instance, for inclusion into the Single Cell Expression Atlas (https://www.ebi.ac.uk/gxa/sc/home), which is a sister resource to ArrayExpress. With new single-cell technologies constantly emerging, new protocols and corresponding annotation vocabularies are being developed, and consequently new metadata fields are required to accurately reflect the data. An increasing number of submitted single-cell sequencing datasets with different protocols and data types has enabled us to collect and standardize this information and implement a user guide for annotating single-cell experiments. This guide helps the submitters to choose the sample metadata requirements for each experiment type and to capture and represent the quality controls. We try to capture the minimal necessary technical information about the cell isolation and library preparation method and, where possible, the type of previously published protocol that was followed. For droplet-based technologies, we capture the information about sample, cell and unique molecular identifier barcodes, such as their location and length, in order to process the sequencing read data accurately and extract information from individual cells and messenger RNA molecules. Throughout the guide, submitters are provided with examples available in ArrayExpress.

## ANNOTARE SUBMISSION TOOL

Over the last year, 99% of the submissions to ArrayExpress came in through our web-based submission tool Annotare. Most of the latest Annotare development was focused on making the submission process as easy as possible for the user, as well as on minimizing time spent by curators on submissions handling and metadata curation.

### Submission templates

**Figure 2.**
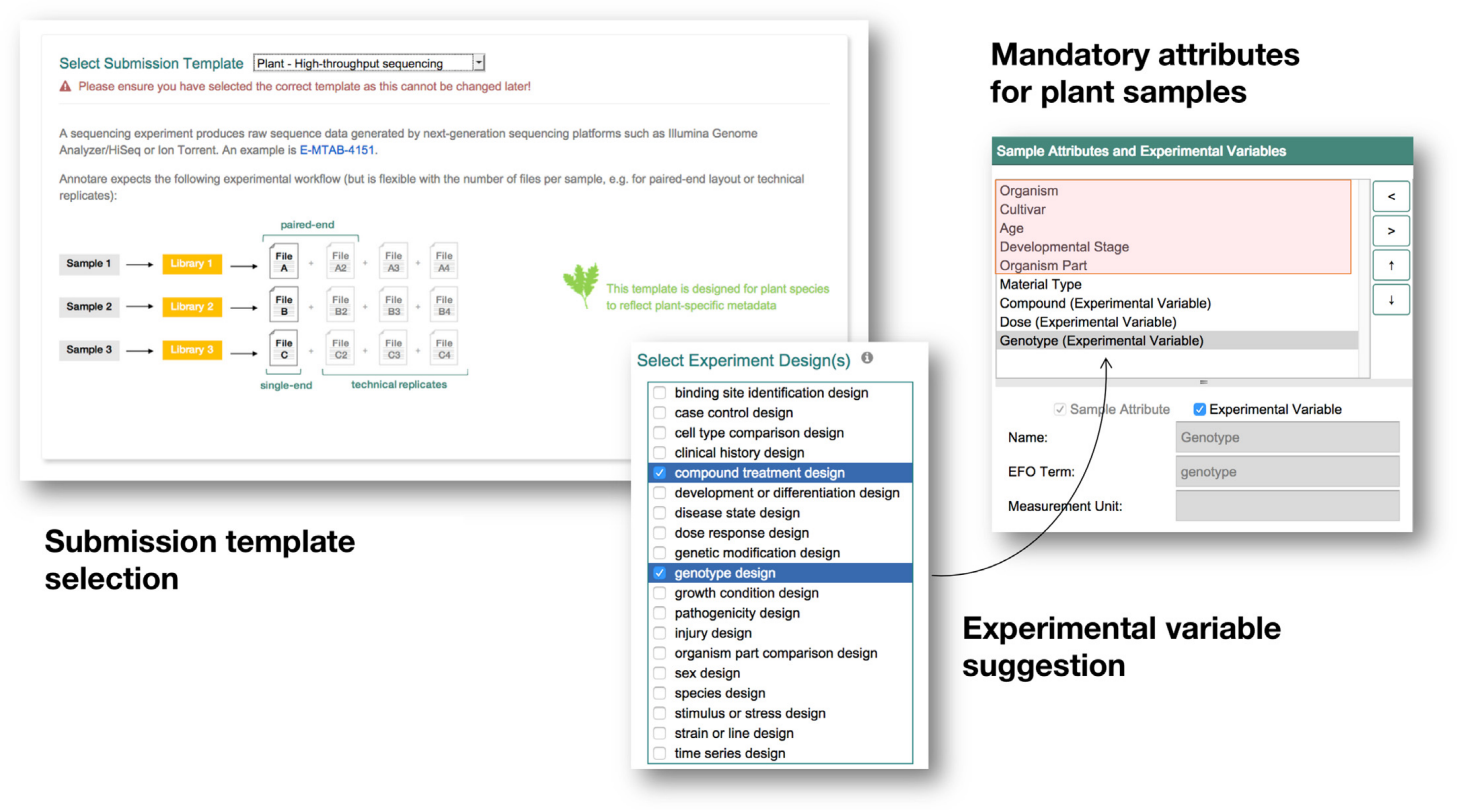
Annotare submission set-up and template selection. First, the user chooses the template type e.g. ‘Plant - high-throughput sequencing’. Then, the study design can be selected from a list of suggestions. Based on these choices, the relevant sample attributes and experimental variables are preselected.

Since Annotare needs to be suitable for the submission of a wide variety of functional genomics experiments, covering all species and experiment designs, ranging from human cell lines to rare marine species, compound treatments and technical designs, mandatory metadata fields need to be kept sufficiently generic to suit any of these experiments. It is thus a challenge to retrieve essential metadata that is specific for a certain experiment design, e.g. the compound name and dose if a compound treatment was performed, without overwhelming the submitter with many questions irrelevant to their particular experiments. As the most recent feature addition to Annotare, we are solving this problem by introducing tailored templates for different experiments, for instance, plant experiments (Figures 1A and 2).

During the submission setup, Annotare asks a number of guided questions and prepares forms with specific fields that are relevant to the experiment at hand. Based on the experiment design type, which the submitter chooses during the submission setup, Annotare suggests an appropriate experimental variable to be included in sample annotation, e.g. ‘time’ for a ‘time series design’. Specifically for plant experiments, mandatory sample attributes, such as organism part, developmental stage and genotype, and a growth protocol are included in the fields that must be filled. We have experienced that these updates greatly improved the metadata quality since their introduction. The metadata requirements were developed to conform with standards that are applied to curate transcriptomics data for Expression Atlas. Thus, this facilitates integrating experiments submitted through Annotare into Expression Atlas.

### User experience changes

**Figure 3.**
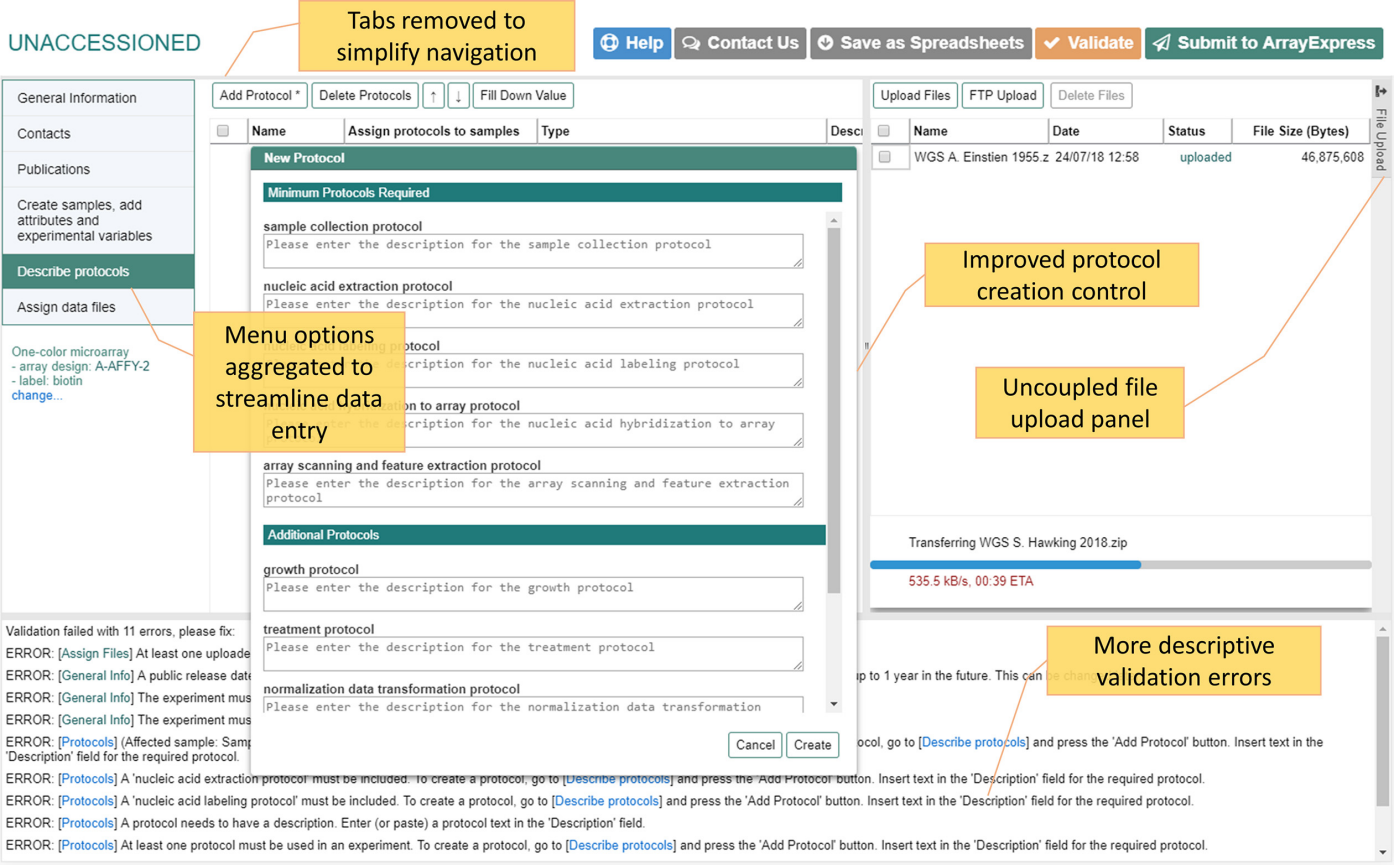
The updated Annotare interface featuring an uncoupled file upload panel, simplified navigation, improved validation error reporting and a new protocol entry panel.

During the past 3 years, there have been multiple improvements in the Annotare interface in order to make the submission process easier and enhance user experience (Figure 3). For instance, we have improved the interface by making the data entry forms more accessible and providing a more streamlined step-by-step approach to data collection. Another major change has been to uncouple the file upload available on every screen. This makes it possible to edit the metadata fields while files are uploading in parallel and in turn, decreases the total time spent in completing a submission. The upload panel allows drag-drop gesture to be used for multiple files and can also be minimized to provide more space for data entry.

To facilitate the navigation for new submitters, we have improved contextual help, adding a short usage message to almost all features and buttons. A video has been added to the home page, which guides new users through the submission process. A ‘Getting Started’ button has been introduced, which points the user to Annotare help pages, with improved easy to follow instructions and screenshots. Validation error messages, which referred to the Investigation Design Format and Sample and Data Relationship Format data files (8) produced by Annotare, have been simplified by aggregating similar messages and adding more descriptive text and tips to help the user in solving the issues as well as links to pages which need updating. Another improvement has been to update the protocol entry dialogue so that all mandatory protocols are added to the experiment upon creation.

### Submission processing

We have automated the sequence file (FASTQ) validation, which has significantly reduced the response time to report any errors in these files and their integrity. Any problems are reported back to the submitter directly after the submission and the affected data files can be uploaded again.

Annotare is now tightly integrated with the EMBL-EBI-wide Request Tracker ticketing system. Whenever users create a new submission, a ticket is created and the submitter is sent an email immediately with the ticket number in the subject line. All correspondence pertaining to that submission is then carried out using the same ticket which helps in streamlining communication, in particular, for users with a large number of concurrent submissions.

Another important process improvement which is largely invisible to the users but affects the daily operations has been to update our infrastructure to allow for parallel processing of incoming submissions. This has allowed an overall increase of throughput of the submission processing module, even though there has been a substantial increase in the number of submissions containing large files.

## OTHER DEVELOPMENTS

Several improvements have been made on the ArrayExpress web application interface in order to facilitate user experience. The experiment detail page has been updated to include links to additional files that submitters have included with the experiments (e.g. a script which they used to process data) but cannot be included as raw/normalized data files. This feature has been left deliberately unstructured to allow submitters more freedom in providing any additional information which may assist in understanding their dataset. As community standards emerge, more structured representation will be implemented. It is also now possible to add links leading to related ArrayExpress experiments, or related data in other EMBL-EBI resources, such as PRIDE (9), ENA (10) and MetaboLights (11). A new search field for ‘sample attribute category’ (shortened as ‘sac’) has been added, which is useful to retrieve all experiments where a certain attribute is present, e.g. all experiments that have ‘age’ specified. A new column has been added to the sample view page to show all assay-related variables, such as assay name and label.

The ArrayExpress application programming interface (API) to programmatically access data and metadata has been updated to version 3 with several new features. Searching and retrieving protocols were enabled e.g. via keyword appearance in the text, protocol type or protocol accession. The new samples search function lets users retrieve detailed information for each sample associated with an experiment as well as information about the sequencing data files hosted at ENA. Also the new search fields for ‘sample attribute category’ has been incorporated into the programmatic experiment search.

Given that Expression Atlas now selects experiments for re-processing and curation directly from Gene Expression Omnibus (GEO), and the pending move of ArrayExpress to BioStudies Database, we have stopped GEO data imports into ArrayExpress. Existing GEO experiments in ArrayExpress will continue to be available for search and download.

Finally, we have also set up a Twitter account with the handle @ArrayExpressEBI and tagged our tweets with hashtag #AnnotareEBI to allow users to track developments and real-time notices of Annotare accessibility.

## FUTURE DIRECTIONS

With the increasing prevalence of single-cell assays, and in particular scRNA-seq experiments, our focus in the next few years will be on constantly improving the ways of capturing and representing these data. Annotare will make it easy to submit scRNA-seq data via specialized single-cell submission templates, collecting all data and metadata necessary for re-analysis of these experiments. This will ensure that scRNA-seq data can be consistently re-processed and the results made available at EMBL-EBI via the integrated Single Cell Expression Atlas, which not only increases the utility of these data, but also serves as a test for their re-usability.

As mentioned in the introduction, given the increasing share of experiments involving multiple technologies assaying multiple aspects of biology, ArrayExpress will be superseded by the BioStudies Database. However, the current data submitters and users of ArrayExpress will experience little change as the functionality of ArrayExpress query interface will be maintained in BioStudies. Gene expression and other functional genomics data relevant to Expression Atlas will be acquired via Annotare and re-processed and loaded into Expression Atlas as before. The BioStudies Database is a new EMBL-EBI resource that aims to package all the data associated with a publication: links to the individual components of multi-omics datasets, unstructured data, ‘orphan’ data (i.e. data that could have defined standards, but they have not yet been developed) and supplementary data. This database accepts a wide range of types of studies described via a simple format, and enables manuscript authors to submit Supplementary Data and link to it from the publication. Its data model, submission services, data rendering capabilities and APIs enable it to receive and publish functional genomics datasets in the MAGE-TAB format (8), allowing the users to get an overview of the experiment structure, similar to the way information is presented in ArrayExpress. The existing ArrayExpress experiment (study) accession numbers will be preserved and grouped under the ArrayExpress Project, and the existing URL links to ArrayExpress experiments will remain valid. We are currently preparing an API migration guide. There will be a transition period where datasets are loaded into ArrayExpress and BioStudies in parallel and we will solicit comments from both data submitters and consumers on data presentation and API access in BioStudies.
